# Case report: Plate-assisted bone transport with uniplanar external fixator in large bone defects of the humerus

**DOI:** 10.1016/j.ijscr.2024.109898

**Published:** 2024-06-14

**Authors:** Deniz Akbulut, Mehmet Coskun, Javad Mirzazada, Arda Berkan Sezgic

**Affiliations:** a Tatvan Can Hospial, Bitlis, Türkiye; b Medistanbul Hospital, Istanbul, Türkiye; c Medical Park Kocaeli Hospital, Kocaeli, Türkiye

**Keywords:** Case report, Humerus large bone defect, Plate assisted bone transport

## Abstract

**Introduction:**

We present a case series of two patients who underwent plate-assisted bone transport (PABT) with a uniplanar external fixator for the treatment of large bone defects of the humerus. The efficacy and outcomes were evaluated.

**Case presentation:**

A retrospective review of patients treated with PABT for humeral defects over a 2-year period was performed. Proper gap healing occurred within 3 months, and a long course of physiotherapy was involved in obtaining satisfactory outcomes.

**Discussion:**

Our results demonstrate that PABT might be a potentially effective alternative for large humeral defects, allowing controlled bone lengthening and healing without disrupting vascularity and providing stability for early motion.

**Conclusion:**

PABT appears to be a viable option for the management of large humeral defects with good functional outcomes and a manageable complication profile.

## Highlights

• Bone transport could be an effective alternative which can be performed with an external fixator alone or combined with an intramedullary nail or plate.
• The bone transport is then initiated by gradual distraction while the rotational, bending, distractive, and compressive forces between two major components are mitigated.
• In current practice, bone transport over an intramedullary nail (BTON) could be biomechanically more favorable in the lengthening of long bones.

## 1 Introduction

Treatment of large bone defects of the humerus remains a challenge. There are a number of techniques for obtaining structurally stable healing, such as isolated shortening, compression–distraction at the fracture site, shortening followed by lengthening in a corticotomy distant from the site, plating with vascularized/non-vascularized fibular structural graft, and segmental bone transport [[1], [2], [3]]. Isolated shortening elicits functional impairment, and compression-distraction is effective, although adequate timing is essential. Corticotomy is a prerequisite for ideal biology, and fibular grafting is technically challenging and associated with stress fractures or nonunion, which are reasons for treatment in the first place.

Bone transport could be an effective alternative which can be performed with an external fixator alone or combined with an intramedullary nail or plate. Plate-assisted bone transport (PABT) offers advantages of controlled distraction and preservation of vascularity. The humerus is a long bone that is well known to sustain isolated shortening with satisfactory results. In this paper, we present a case series of two patients who underwent PABT with a uniplanar external fixator for the treatment of large bone defects of the humerus.

## 2 Case presentation

The two patients were males aged 38 and 45 years. They each had large humeral defects that were treated with PABT using a uniplanar external fixator between 2021 and 2023 in Van Regional Teaching Hospital of the Turkish Ministry of Health. Both cases had oligotrophic non-unions.

The mean follow-up time was 24 months. Patient demographics, surgical details, and radiographic and functional outcomes were recorded. The functional outcomes included the range of motion, Constant score, and DASH score. Consecutive radiographs were used to assess bone regeneration and final healing.

### 2.1 Case 1

The first patient was a heavy smoker with a history of 20 pack-years and never quit even throughout our treatment. His right humerus was fractured in a motorcycle accident, and he was admitted and treated elsewhere. The first attempt at fracture management was conservative and failed in 4 months. Treatment with a dynamic compression plate (DCP) was planned immediately after, which failed in 5 months. Exchange plating and fibular allografting were attempted, but there were no signs of callus formation in 3 months, and the patient had a huge deal of pain and dysfunction from where our treatment started.

The patient was unable to lift his limb to eye level without contralateral arm support, so core muscles were atrophied, and chronic regional pain was present. The patient was on an aggressive regimen of pain medication. Hand and wrist functions were rather preserved. Unfortunately, the radiological documentation from the previous interventions was unavailable.

### 2.2 Case 2

The second patient was also a heavy smoker with a history of 30 pack-years and never quit even throughout our treatment. His left humerus had an open fracture from an extravehicular traffic accident. Treatment was started elsewhere. During 3 months of follow-up, osteomyelitis of the humerus flared up. *Staphylococcus aureus* was isolated when the patient was admitted. He had mild to moderate pain and could lift his arm up to the eye level without difficulties but with significant scapulothoracic involvement.

The most prominent complaint was pus drainage with a need to change dressings twice daily. His white blood cell (WBC) count was normal, his C-reactive protein (CRP) level was significantly elevated, and he had no fever. Intensive antibiotic therapy was applied right away. Debridement, bone cementing, and external fixation were performed. Cementing provided an extra advantage of membrane induction. Pus drainage never occurred again, and after 2 months, his CRP level returned to normal according to three consecutive measurements. PABT was applied immediately afterward.

### 2.3 Surgical technique

PABT with a uniplanar external fixator in the femur and tibia is a well-explained technique. We adapted the same concept to the humerus. First, a precalculated adequate amount of bone is resected through a lateral arm incision. The bone block to be transported is then osteotomized and meticulously distracted through the corticotomy from the proximal portion of the shaft. Proximal and distal portions are fixated with a locking plate.

An external fixator is applied to the humerus and the bone block. The bone transport is then initiated by gradual distraction while the rotational, bending, distractive, and compressive forces between two major components are mitigated. The bone transport is continued until the desired length and alignment are achieved. Finally, the fixator is removed, and the transported bone block is stabilized using additional screws. Ultimate care is taken not to damage the radial nerve. The induced membrane technique (Masquelet) [1,4] was used in the second case in order to achieve proper callus, so the patient had a single additional intervention prior to PABT surgery.

## 3 Results

Absolute bone consolidation was achieved at an average of 9.5 months (patient 1: 10.5 months; patient 2: 8.5 months). Fixators were removed at an average of 54 days (patient 1: 60 days; patient 2: 48 days) (Fig. 1, Fig. 2, Fig. 3, Fig. 4, Fig. 5, Fig. 10, Fig. 11, Fig. 12, Fig. 13, Fig. 14). Both patients had functional range of motion for daily activities and intensive physical therapy following successful union (Fig. 6, Fig. 7, Fig. 8, Fig. 9, Fig. 15, Fig. 16, Fig. 17, Fig. 18). The mean Constant score improved from 35 to 82, and the mean DASH score improved from 65 to 18. The patients achieved good functional outcomes and sturdy bone healing at both the distraction and docking sites.

**Fig. 1:**
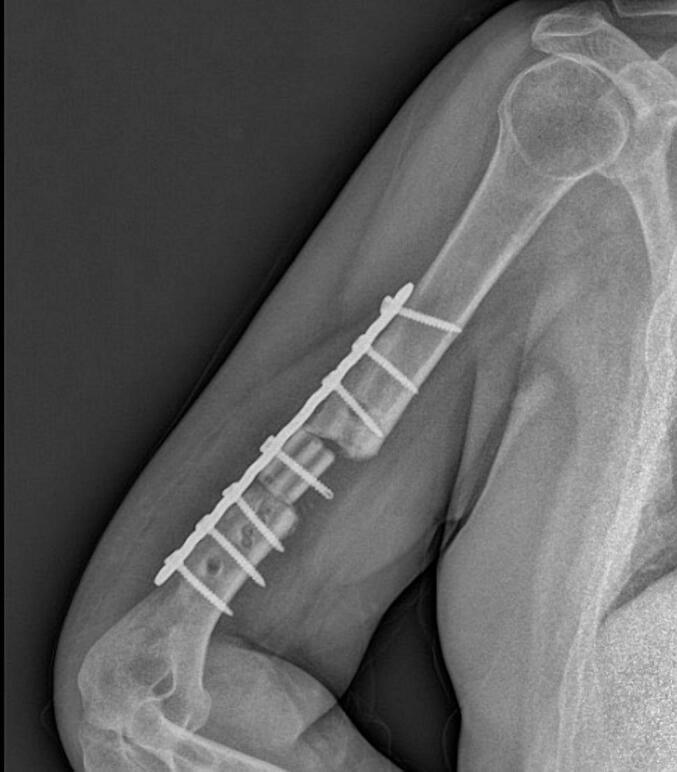
Preoperative assessment, after grafting, before PABT.

**Fig. 2:**
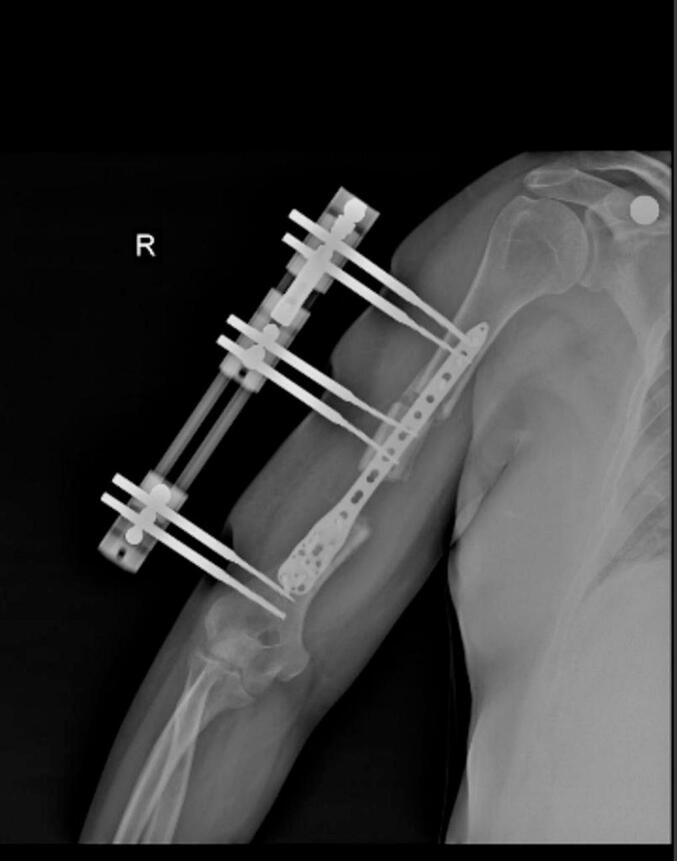
Early postoperative assessment.

**Fig. 3:**
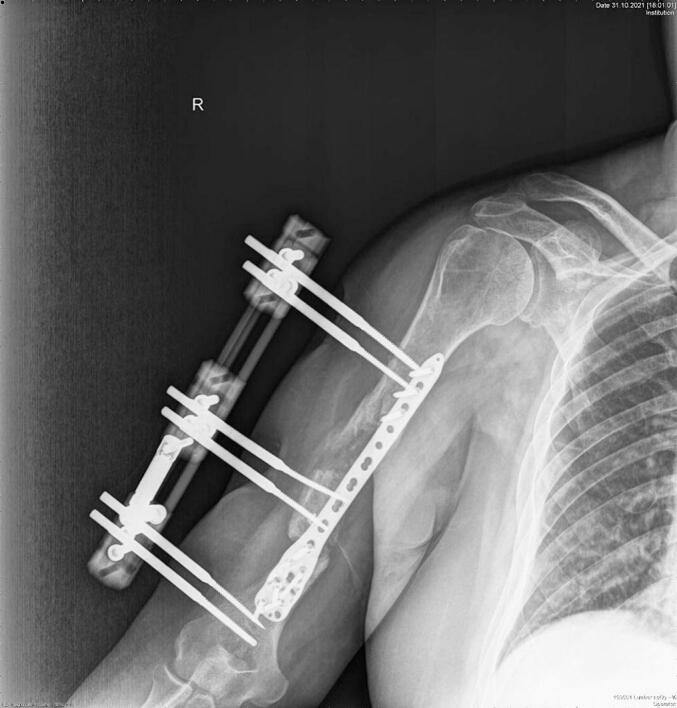
Postoperative assessment, after docking, before external fixator removal.

**Fig. 4:**
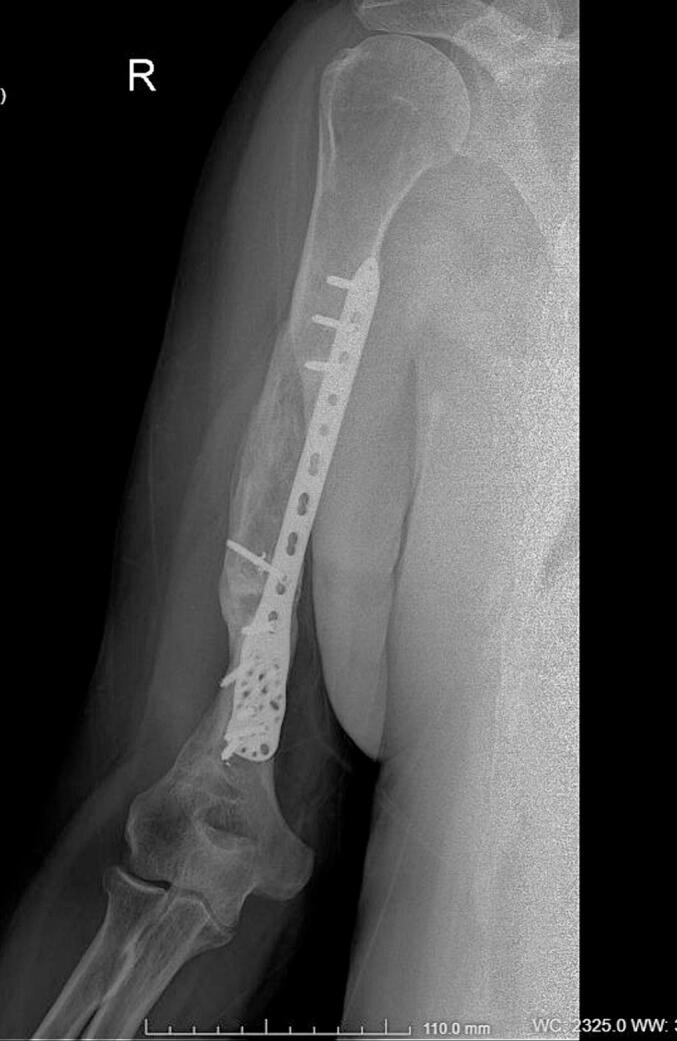
Late postoperative assessment, successfully healed (AP view).

**Fig. 5:**
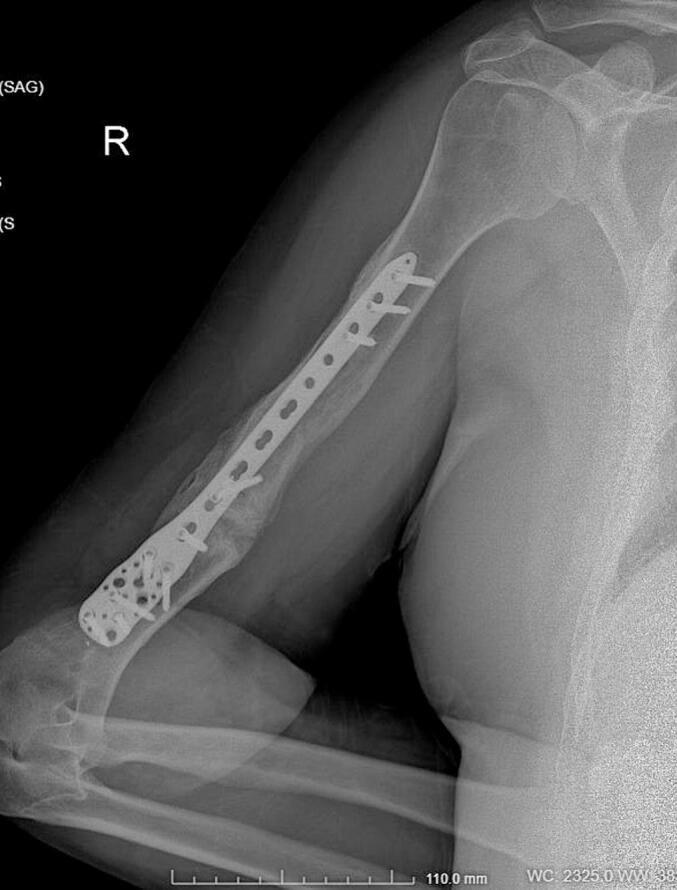
Late lateral postoperative assessment, successfully healed (Lateral view)

**Fig. 6:**
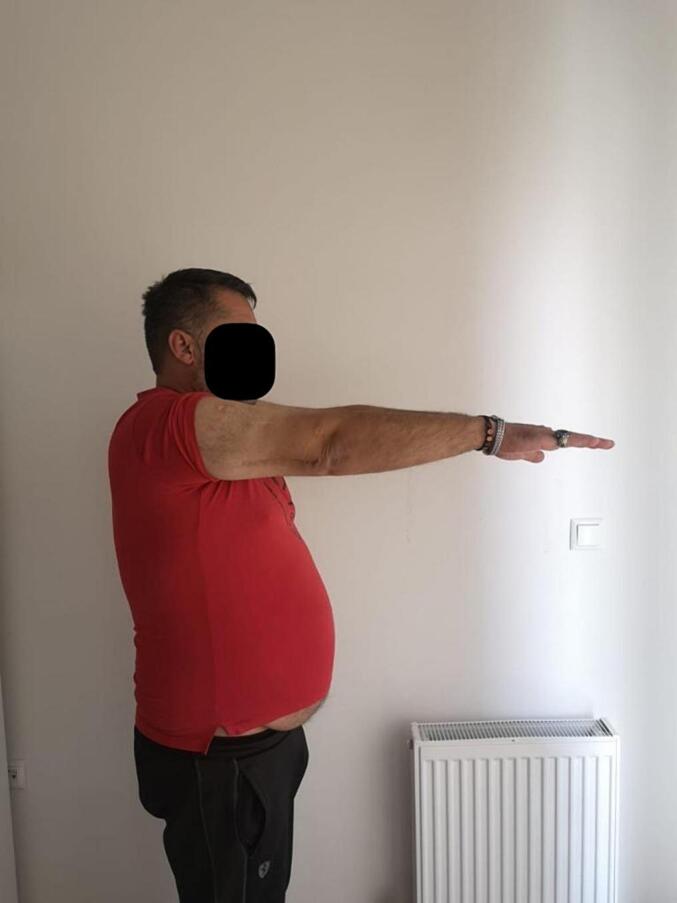
Late postoperative clinical assessment, note the elbow extension.

**Fig. 7:**
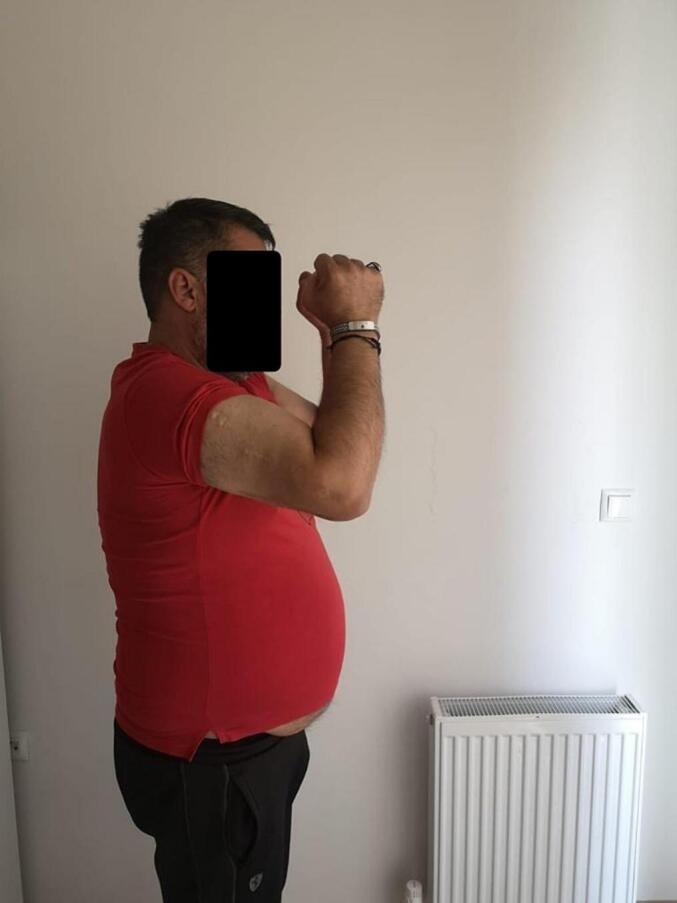
Late postoperative clinical assessment, note the elbow flexion.

**Fig. 8:**
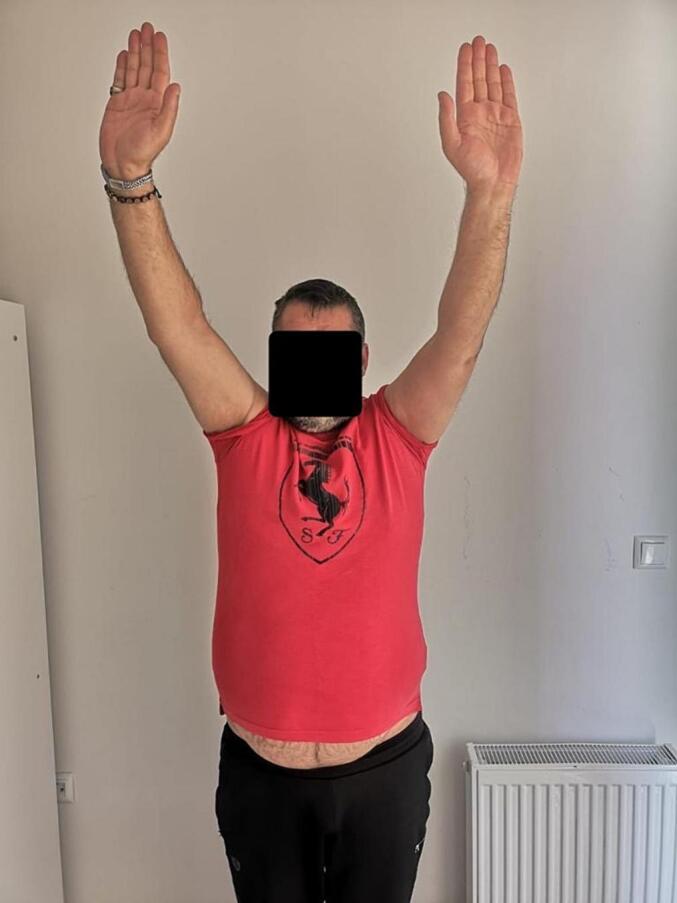
Late postoperative clinical assessment, note the shoulder elevation.

**Fig. 9:**
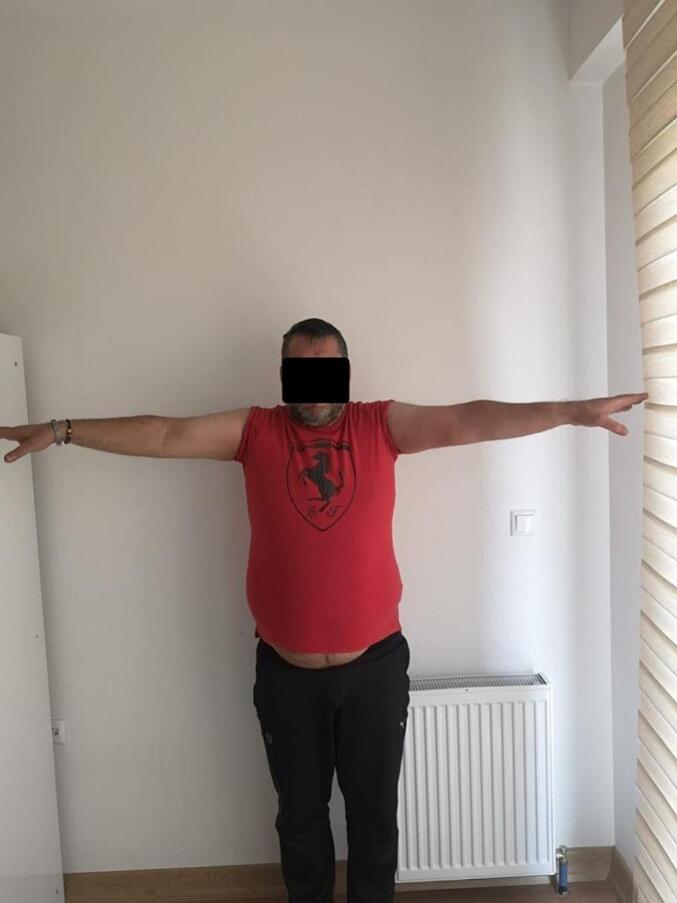
Late postoperative clinical assessment, note the shoulder abduction.

**Fig. 10:**
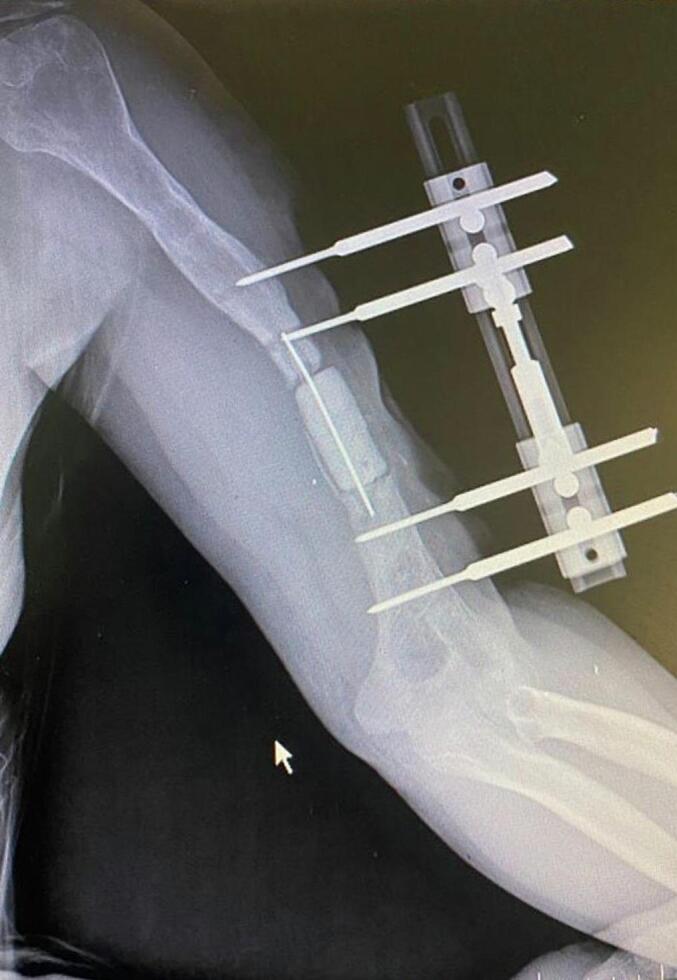
Preoperative assessment, after Masquelet, before PABT.

**Fig. 11:**
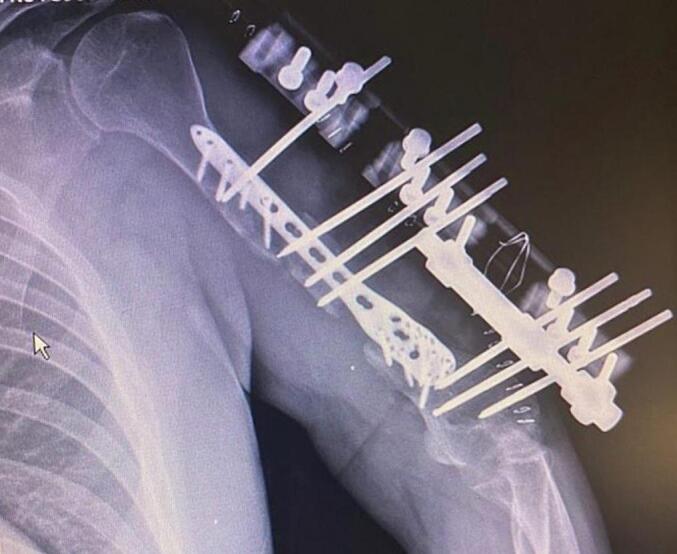
Early postoperative assessment.

**Fig. 12:**
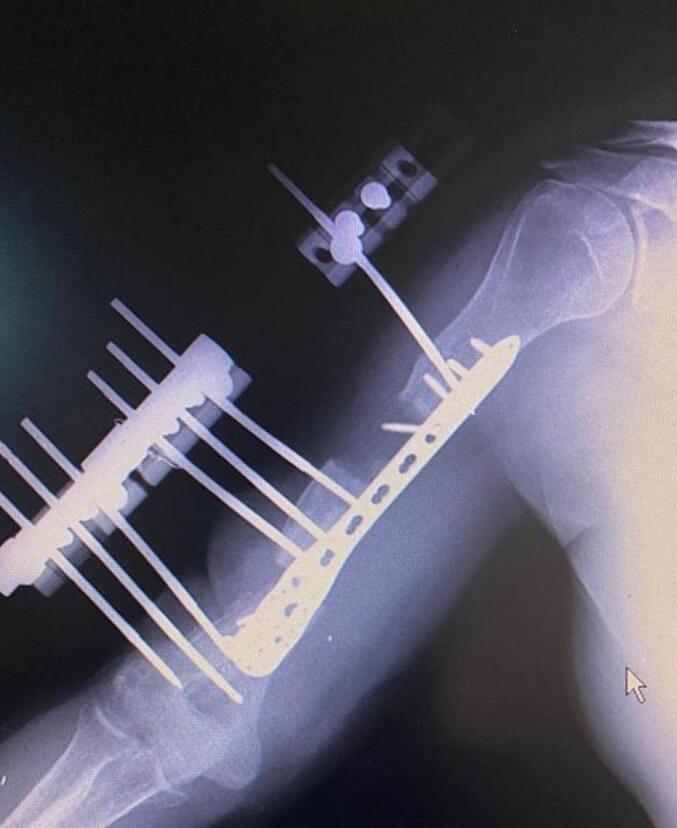
Postoperative assessment, the docking is about to be finalized.

**Fig. 13:**
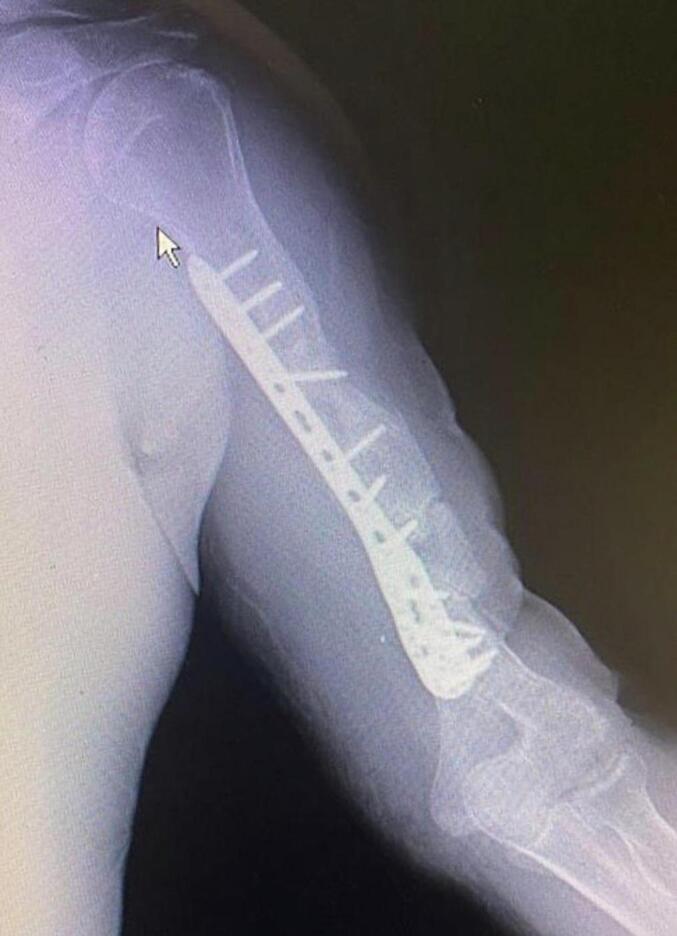
Late postoperative assessment, docking and locking finalized.

**Fig. 14:**
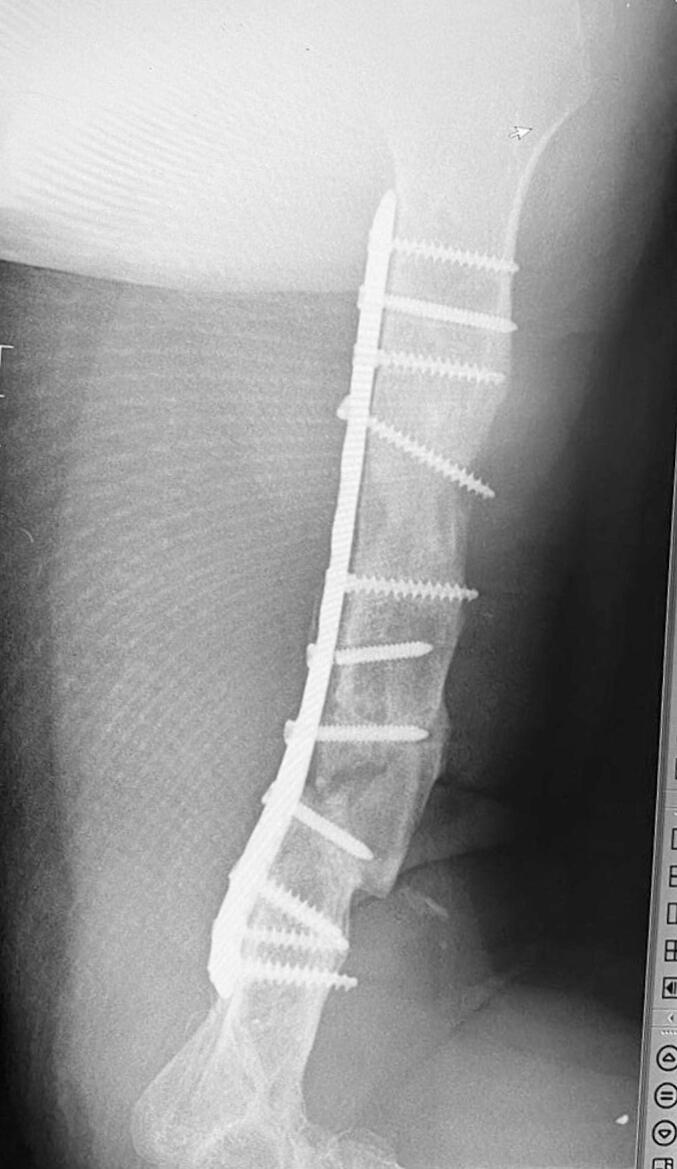
Late postoperative assessment, note the proper healing in closer view.

**Fig. 15:**
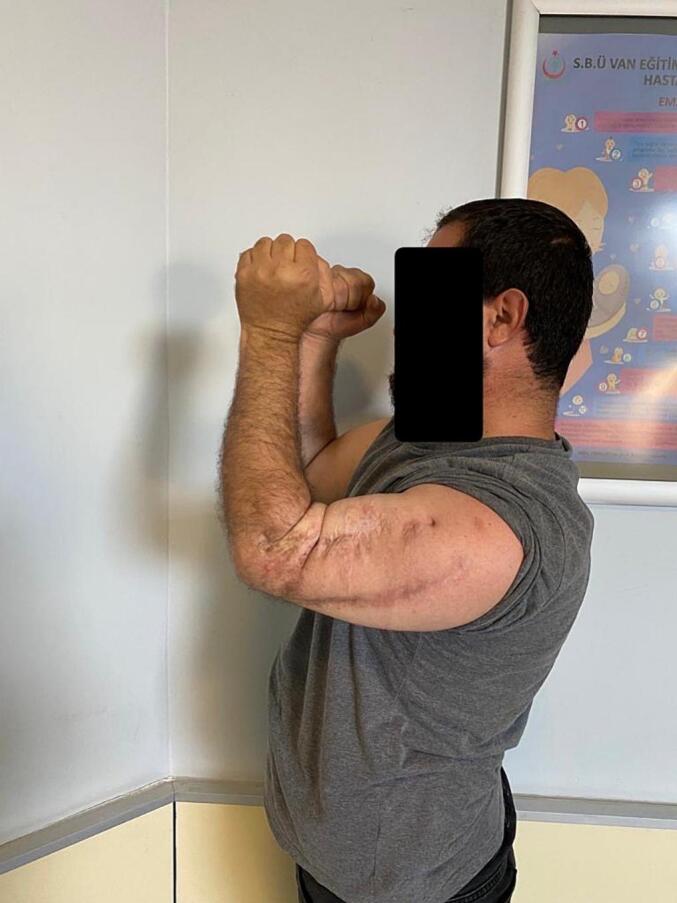
Late postoperative clinical assessment, note the mild elbow stiffness due to recurrent surgeries and delayed healing.

**Fig. 16:**
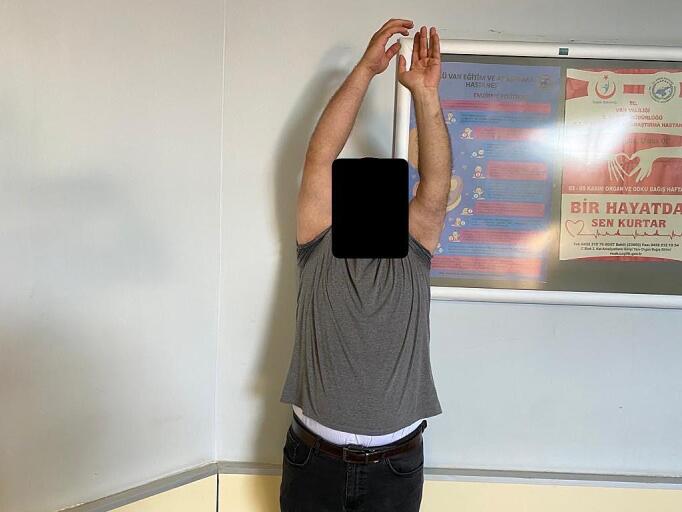
Late postoperative clinical assessment, note the shoulder elevation.

**Fig. 17:**
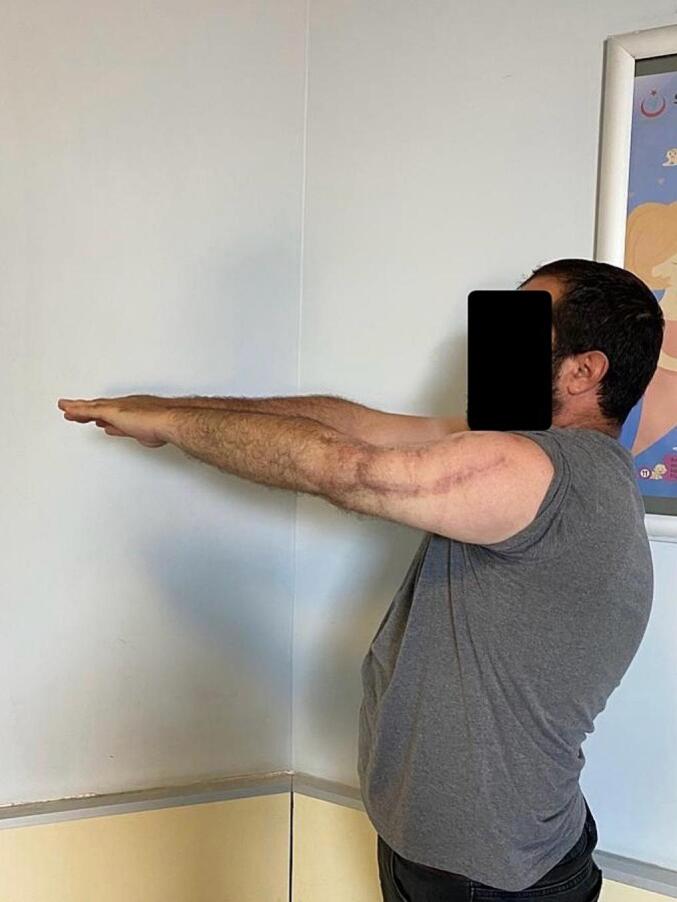
Late postoperative clinical assessment, note the elbow extension.

**Fig. 18:**
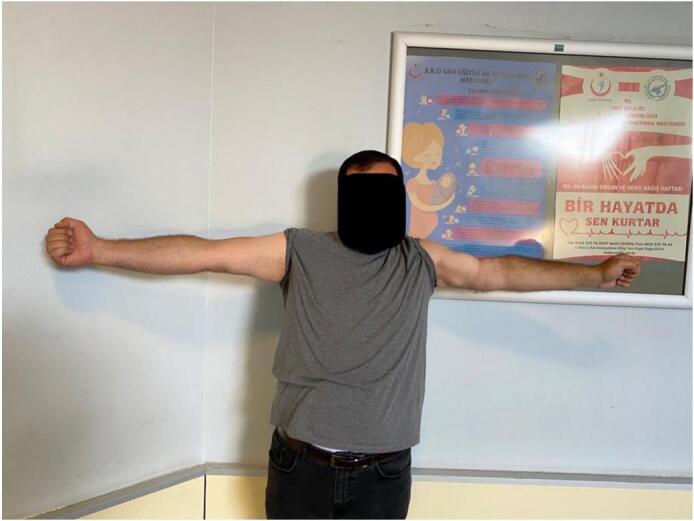
Late postoperative clinical assessment.

The technique allowed for the restoration of bone length and alignment, which is crucial for maintaining proper limb function. The average defect size was 4.8 cm (patient 1: 5.3 cm; patient 2: 4.3 cm). The mean distance of the transported bone was 4.4 cm (patient 1: 5 cm; patient 2: 3.8 cm). The average external fixator index (EFI) was 11.3 days/cm (patient 1: 11.3; patient 2: 11.2), and the average healing index (HI) was 60.2 days/cm (patient 1: 60.3 days/cm; patient 2: 60.2 days/cm) according to the calculation methods reported by Oh et al. [[5], [6], [7]]. There were no instances of pin tract infections, nerve palsies, skin-coverage problems, vascular injuries, distraction-induced vascular dysfunctions, premature consolidation, bone resorption, fracture, or any type of structural insufficiencies whatsoever.

## 4 Discussion

Our results demonstrate that PABT might be a potentially effective alternative for large humeral defects and allows controlled bone lengthening and healing. The outcomes compare favorably to those of other techniques reported in the literature. The advantages include preservation of vascularity, stability for early motion, and high union rates.

According to previous papers, the main indication for bone-transport surgery is a defect size exceeding 10 cm [2,3,8]. Nevertheless, current research is encouraging even for the minor defects [5,[9], [10], [11], [12], [13], [14], [15]]. In current practice, bone transport over an intramedullary nail (BTON) could be biomechanically more favorable in the lengthening of long bones because of the convenient control of the axial alignment. However, there is a possibility of sagittal imbalance or procurvatum deformity in the lower extremities [16], although this might be a minor issue in the upper extremities.

Although our cases had not demonstrated any prominent complications, a brief discussion on this topic would be pertinent. Infection is a significant concern, particularly in cases involving large bone defects and open fractures. Proper surgical technique, including thorough debridement and antibiotic prophylaxis, is crucial in minimizing the risk of infection before starting PABT treatment. Other complications may include pin tract infections, nerve or vascular injury, and joint stiffness. Close monitoring and appropriate management of these complications are essential for optimizing patient outcomes [17].

Despite the demonstrated effectiveness of this method, a large number of cases are necessary to determine the yield success, which is still unclear. Thus, further investigations are indispensable. This work has been reported in line with the SCARE criteria [18].

## 5 Conclusion

This case series suggests that PABT is a viable option for reconstruction of large humeral defects. The method has produced satisfactory bone healing and functional recovery. Nevertheless, further research with larger sample sizes is needed to validate our findings.

## Informed consent

Written informed consent was obtained from the patients for publication and any accompanying images. A copy of the written consent form is available for review upon request.

## Ethical approval

Case report does not require ethical approval in our institution.

## Funding

None.

## Author contribution

All the effort was accomplished by the given authors equally.

## Guarantor

The first given author, Deniz Akbulut, is otherwise the mere guarantor in this study.

## Research registration number

None.

## Declaration of competing interest

None.

## References

[1] Rigal S., Merloz P., Le Nen D., Mathevon H., Masquelet A.C. (2012). Bone transport techniques in posttraumatic bone defects. Orthop. Traumatol. Surg. Res..

[2] Sen C., Kocaoglu M., Eralp L., Gulsen M., Cinar M. (2004). Bifocal compression-distraction in the acute treatment of grade III open tibia fractures with bone and soft-tissue loss: a report of 24 cases. J. Orthop. Trauma.

[3] Paley D., Catagni M.A., Argnani F., Villa A., Benedetti G.B., Cattaneo R. (1989). Ilizarov treatment of tibial nonunions with bone loss. Clin. Orthop. Relat. Res..

[4] Spitler C.A. (2020). Treatment of a segmental defect in the humerus with induced membrane technique. J. Orthop. Trauma.

[5] Park K.H., Oh C.W., Kim J.W. (2021). Matched comparison of bone transport using external fixator over a nail versus external fixator over a plate for segmental tibial bone defects. J. Orthop. Trauma.

[6] Oh C.W., Song H.R., Roh J.Y. (2008). Bone transport over an intramedullary nail for reconstruction of long bone defects in tibia. Arch. Orthop. Trauma Surg..

[7] Oh C.W., Apivatthakakul T., Oh J.K. (2013). Bone transport with an external fixator and a locking plate for segmental tibial defects. Bone J. Joint.

[8] Aktuglu K., Erol K., Vahabi A. (2019). Ilizarov bone transport and treatment of critical-sized tibial bone defects: a narrative review. J. Orthop. Traumatol..

[9] Sen C., Demirel M., Sağlam Y., Balcı H.I., Eralp L., Kocaoğlu M. (2019). Acute shortening versus bone transport for the treatment of infected femur non-unions with bone defects. Injury.

[10] Ferreira N., Tanwar Y.S. (2020). Systematic approach to the management of post-traumatic segmental diaphyseal long bone defects: treatment algorithm and comprehensive classification system. Strateg. Trauma Limb Reconstr..

[11] Olesen U.K., Nygaard T., Prince D.E. (2019). Plate-assisted bone segment transport with motorized lengthening nails and locking plates: a technique to treat femoral and tibial bone defects. J. Am. Acad. Orthop. Surg. Glob. Res. Rev..

[12] Barawi O.A.R. (2016). Management of bone defect of humerus by Ilizarov method. Genij Ortopedii.

[13] Litvina E.A., Semenistyy A.A. (2020). A case report of extensive segmental defect of the humerus treated with Masquelet technique. J. Shoulder Elb. Surg..

[14] Abulaiti A., Liu Y., Cai F. (2022). Bone defects in tibia managed by the bifocal vs. trifocal bone transport technique: a retrospective comparative study. Front. Surg..

[15] Kesemenli C., Subasi M., Kirkgoz T., Kapukaya A., Arslan H. (2001). Treatment of traumatic bone defects by bone transport. Acta Orthop. Belg..

[16] Ferreira N., Saini A.K., Birkholtz F.F., Laubscher M. (2021). Management of segmental bone defects of the upper limb: a scoping review with data synthesis to inform decision making. Eur. J. Orthop. Surg. Traumatol..

[17] Feng D., Zhang Y., Jia H. (2023). Complications analysis of Ilizarov bone transport technique in the treatment of tibial bone defects–a retrospective study of 199 cases. BMC Musculoskelet. Disord..

[18] Sohrabi C., Mathew G., Maria N., Kerwan A., Franchi T., Agha R.A. (2023). The SCARE 2023 guideline: updating consensus surgical CAse REport (SCARE) guidelines. Int. J. Surg..

